# Reactive oxygen species in cancer: Mechanistic insights and therapeutic innovations

**DOI:** 10.1016/j.cstres.2025.100108

**Published:** 2025-08-05

**Authors:** Ning Ma, Yang Wang, Xin Li, Meiling Xu, Dandan Tan

**Affiliations:** 1 Department of Critical Care Medicine, Heilongjiang Provincial Hospital, Harbin, Heilongjiang, 150036, China; 2 Department of Clinical Laboratory, Third Affiliated Hospital, Heilongjiang University of Chinese Medicine, Harbin, Heilongjiang, 150030, China; 3 Department of Critical Care Medicine, The Second Affiliated Hospital of Harbin Medical University, Harbin, Heilongjiang, 150000, China; 4 Department of Nursing, Heilongjiang Provincial Hospital, Harbin, Heilongjiang, 150036, China

**Keywords:** Carcinogenesis, Metastasis, Oxidative stress, Redox balance, ROS, TME

## Abstract

Reactive oxygen species (ROS), once considered mere metabolic byproducts, are now recognized as crucial elements in the complex behavior of cancer, influencing both its progression and vulnerabilities. In healthy cells, ROS maintains a delicate balance: while small amounts are essential for signaling, excessive quantities can cause damage. Cancer disrupts this equilibrium, leveraging ROS to promote proliferation, metastasis, and survival, while employing antioxidant defenses to prevent self-destruction. It is the balance of ROS that is key to cancer growth: as they initiate cancer-related processes such as Mitogen-Activated Protein Kinase (MAPK), PI3K/Akt, and c-Jun N-terminal Kinase (JNK) pathways, and induce inflammation through NF-κB. Additionally, matrix metalloproteinases (MMPs) and vascular endothelial growth factor (VEGF) break down tissue barriers, fostering a tumor microenvironment (TME) conducive to cancer spread. However, this dependence on ROS presents a dual challenge. The timing, location, and quantity of radical formation, along with the surrounding cellular environment, determine whether ROS facilitate cancer progression or lead to cancer cell death. Disrupting this delicate balance of ROS may reveal new treatment methods, transforming cancer's survival mechanisms into significant weaknesses. This study explores the dual roles of ROS in cancer, examining how their contrasting effects impact tumor growth and revealing unexpected opportunities to shift the balance from growth to vulnerability.

## Graphical abstract

**Figure:**
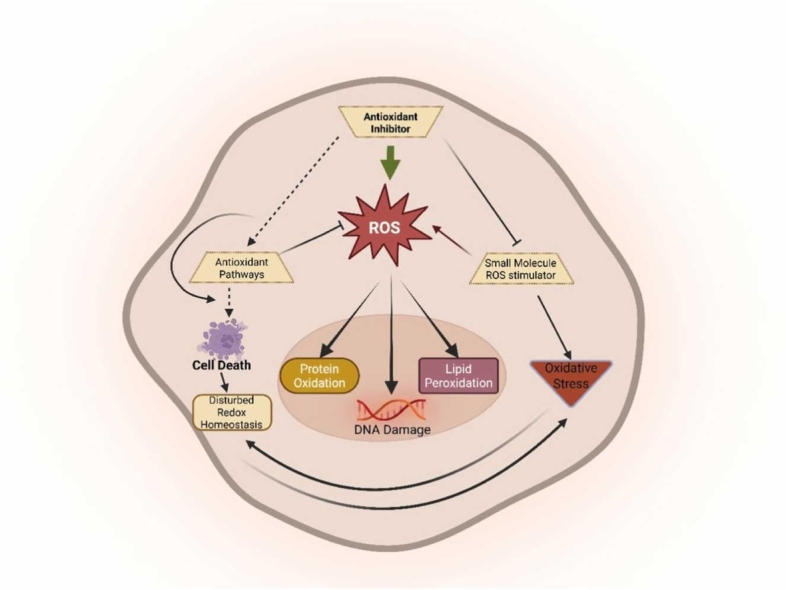
The role of ROS in the treatment of cancer.

## Background

A diverse range of oxygen-holding fragments with unexpected characteristics, often produced as secondary products of biological metabolism, are called reactive oxygen species (ROS).^1^ The majority of ROS contain hydroxyl radicals (•OH) and superoxide (O_2_•-), in addition to singlet oxygen (1 O_2_) and hydrogen peroxide (H_2_O_2_), which is non-radical.^2^ ROS can regulate several biological processes. Disruptions in the balance of redox reactions, characterized by an overabundance or deficiency in the generation of ROS, have detrimental consequences and are linked to several clinical disorders.^3^ It has conclusively shown that ROS significantly determines several aspects of cancer advancement, such as the development of cancer, the growth of tumors, invasion, angiogenesis, metastasis, and resistance to treatment.^4^ Resistance to drugs is the primary impediment to the recovery of several cancer patients. Collected information has revealed many molecular mechanisms that contribute to resistance to cancer therapy. Tumor variables include several characteristics, including cancer stem cell (CSC) plasticity, oncogenic mutations, epigenetic alterations, tumor heterogeneity, and interactions between the tumor and its host, such as the immune system and the surrounding environment.^5^ The production of ROS is a crucial factor in the therapeutic benefits of commonly prescribed chemotherapeutic drugs.^6^ However, cancer cells develop resistance to chemotherapy treatments because of their capacity to adapt to natural or drug-induced oxidative stress.^7^ Gaining insight into the molecular mechanisms liable for treatment resistance in individuals may allow for specifically targeting ROS systems as a therapeutic approach. This review analyses the purposes of ROS in cancer. These include ROS's participation in chemoresistance mediated by transcription factors, its contribution to the maintenance of CSC, and its influence on the tumor microenvironment (TME). We investigate the feasibility of using the ROS system as a tactic to surmount resistance to cancer treatment.

## ROS

**Fig. 1:**
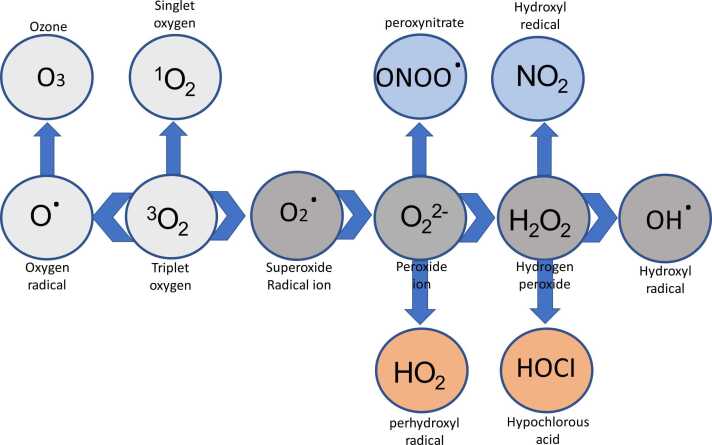
A diagram illustrates the fundamental method of producing ROS.

ROS are produced by a reaction between oxygen molecules (O_2_) and their byproducts with different molecules (Figure 1). ROS may be categorized into two distinct groups: radicals and non-radicals. This classification is determined by whether the ROS possesses unpaired electrons. The energy-impelled arrangement of the electrons in O_2_ produces two singlet states, namely sigma and delta, which are chemically reactive oxygen forms.^8^ As a replacement, O_2_ undergoes a sequence of electron incorporations to produce peroxide ion (O_2_^2^), superoxide anion (O_2_•), and oxide ions (O_2_). Various oxidases, such as those present in the xanthine oxidase (XO), mitochondrial electron transport chain (ETC), Cytochrome P450 (CYP), and nicotinamide adenine dinucleotide phosphate (reduced) (NADPH) oxidases (NOX), produce O_2_•, a rather moderate oxidant radical, as their primary outcome of oxidation. Because of its non-radical characteristics, H_2_O_2_ has a more oxidizing ability and a longer half-life in comparison to O_2_. Monooxygenases and oxidases produce hydrogen peroxide (H_2_O_2_), which moves into H_2_O by the catalase (CAT) or glutathione peroxidase (GPX).^9^ The •OH is the most highly reactive form of oxygen. The formation occurs via the reaction between H_2_O_2_ and transition metals, such as iron and copper. For instance, singlet oxygen (1 O_2_) only targets guanine, whereas HO• can oxidize all components of deoxyribonucleic acid (DNA). ROS may lead to the oxidation of protein sulfhydryl (-SH) groups, resulting in lipid peroxidation. Within the group of ROS, the •OH has an increased reactivity towards amino acid residues. Oxygen (O_2_) and H_2_O_2_ have been scientifically shown to contain significant biological and signaling qualities.^10^ They have greater reactivity than O_2_ but lesser reactivity than HO•. Different metabolic enzymes containing iron-sulfur clusters have been shown to undergo oxidation and subsequent inactivation due to O_2_•, resulting in the liberation of iron and the generation of HO•. The primary origins of ROS in human cells include the mitochondrial ETC, NOX enzymes, and the generation of H_2_O_2_ during protein folding in the endoplasmic reticulum (ER). Around 1% of oxygen experiences premature oxygen reduction, resulting in the formation of O_2_•, due to electron escape in complexes I and III of the ETC.^11^ By contrast, the cytochrome C oxidase complex converts 95% of oxygen into H_2_O throughout adenosine triphosphate (ATP) synthesis by oxidative phosphorylation in mitochondria. An increased mitochondrial transmembrane potential acts as an obstacle to electron transport and substantially diminishes the functioning of complexes I and III, resulting in reduced rates of respiration. Therefore, this indicates an expanded creation of ROS inside the mitochondria. Manganese superoxide dismutase (SOD), copper/zinc SOD, and other antioxidants, ie, peroxiredoxins and peroxidases found in the mitochondrial matrix, convert oxygen inside the mitochondria into H_2_O_2_.^12^ tumor cells may alter the creation of ROS and their function to mitigate oxidative stress to facilitate the development of malignancy.^13^ The association between high expression of ROS emanating from mitochondria and extra sources and the progression of malignancy in various types of cancer. NOX protein is found in several intracellular structures, including mitochondria, ER, nuclear or plasma membranes, invadopodia, and focal adhesions. The generation of O_2_ is solely attributed to the transfer of a single electron from NADPH to molecular oxygen.^14^ Dual oxidases (DUOX1-2) and NOX (NOX1-5) include a total of seven distinct catalytic isoforms, which display a wide range of biochemical, structural, and subcellular localizations. Oxygen molecules (O_2_) may be transformed into H_2_O_2_ by the activity of closely associated enzymes like SOD, NOX like DUOX 1-2, and human NOX4 are through a spontaneous reaction.^15^ Phagocytes lead to the production of ROS. The process is facilitated by several isoforms of NOX and oxidases.^8^ NOX1 is linked to the advancement and protection of cancer characteristics via the generation of extracellular O_2_. The intercellular route for ROS signaling is facilitated by the generation of hypochlorous acid (HOCl), singlet oxygen (1 O_2_), •OH, and peroxynitrite (ONOO-) from H_2_O_2_ and superoxide (O_2_•).^16^ This work primarily investigates the signaling pathways that are modulated by intracellular ROS. Various oxidases are found in diverse parts of cells, such as cell membranes, peroxisomes, or the ER. These oxidases also produce molecular oxygen (O_2_•) and H_2_O_2_.^17^ Interleukin-1, Tumor necrosis factor-alpha, tumor promoter 4-o-tetradecanoylphorbol-13-acetate (TPA), and the bacterial lipopolysaccharide induce the activation of cyclooxygenase (COX), which leads to the production of ROS.^8^ O_2_• is formed when an activated form of O_2_ is released without changing the substrate, whereas H_2_O_2_ is created when an electron is released during the protonation of the reaction peroxycytochrome P450. Importantly, the oxidation processes carefully control the activity of each of these enzymes that generate ROS.^18^ Ascorbic acid, reduced flavins, and catecholamines have directly reacted with oxygen, leading to the production of O_2_•.^10^ The process of autooxidation in iron-binding proteins is crucial for immune response and iron metabolism.^19^ Oxidative stress is the alteration of the balance of oxidants and antioxidants, with a bias towards the oxidants. This scenario has the potential to cause changes in redox signaling, as well as harm to proteins, phospholipids, and nucleic acids, and perhaps result in cell death.^20^ SODs are a class of compounds that catalyze the conversion of O_2_• into molecular oxygen and H_2_O_2_. There are three distinct forms of SOD enzymes: cytoplasmic, mitochondrial, and extracellular. Afterward, the former is either transformed into H_2_O by GPX, which enables the oxidation of glutathione (reduced) (GSH) to glutathione disulfide (oxidized) using H_2_O_2_,^20^ or it is metabolized into H_2_O and O_2_ by CAT. These results highlight the significance of O_2_• scavenging. Heterozygous SOD2 genotype exhibited increased cancer occurrence, greater levels of nuclear DNA oxidation, and accelerated mitochondrial oxidative damage throughout their whole lives. In addition, SOD1 deficiency showed a vulnerability to cancer, including liver cancer, along with hearing loss and cognitive decline.^21^ It is important to highlight that the antioxidant system may be affected by ROS, which can cause the oxidation and loss of activity of both SOD and CAT in the presence of molecular O_2_. CAT is essential in regulating Reactive Nitrogen Species by oxidizing •NO or breaking down ONOO- into nitrite (NO_2_).^16^

## Source and control of ROS formation

ROS serve as vital signaling molecules at low levels but become destructive when uncontrolled. Cellular health hinges on maintaining this equilibrium through precise synthesis, scavenging, and repair mechanisms.

### Origin of ROS

The primary origins of endogenous ROS are in mitochondria, peroxisomes, nicotinamide adenine dinucleotide oxidases (NOX), and the ER. During respiration, electron leakage inside mitochondria results in the conversion of around 1%-2% of ingested O_2_ into O_2_•. This mechanism is the initial origin of endogenous ROS. Superoxide is mostly produced when electron transfer occurs in complex I and complex III of the mitochondrial ETC, respectively.^22^ Mitochondrial outer membrane proteins, monoamine oxidases, can increase the internal reservoir of ROS·H_2_O_2_ is produced as a byproduct when monoamine oxidases use the flavin adenine dinucleotide cofactor to aid in the oxidative breakdown of monoamines.^23^ NOXs, a distinguished cluster of enzymes responsible for producing ROS, were initially known in phagocytic cell membranes. The NOX isoforms, including the NOXs, consist of a catalytic core composed of six transmembrane domains, binding sites for flavin adenine dinucleotide and NADPH, and two heme-binding regions. NADPH transfers electrons to molecular oxygen via NOXs, majorly to the generation of superoxide and H_2_O_2_.^24^ Cellular components such as the peroxisomes, ER, and enzymes CYP0, COX, and XOs may produce exogenous ROS.^25^ In addition, the ROS produced from lipids has lately drawn attention due to the effect of redox signaling. Moreover, different variables, ie, radiation, medications, foreign substances, smoking, pollution, ultraviolet radiation, and tobacco, all have a role in generating ROS outside of cells. The main locations where cellular ROS are produced are the mitochondrial ETC, NOX complex, peroxisomes, and ER. Inside mitochondria, O_2_ is converted into a very ROS (O_2_•) because electrons are released from the ETC.^26^

### Mechanism of ROS

ROS are chemically unstable molecules, including superoxide (O₂⁻), hydrogen peroxide (H₂O₂), and hydroxyl radicals (OH·), generated through the partial reduction of oxygen (Figure 1). Their formation begins primarily in mitochondria, where electrons “leak” from the ETC during ATP synthesis, reacting with oxygen to form superoxide. NOX enzymes in cell membranes deliberately produce ROS as signaling molecules or antimicrobial agents by transferring electrons to oxygen. Peroxisomes contribute through fatty acid β-oxidation, releasing H₂O₂ as a byproduct, while ER stress generates ROS during protein folding via disulfide bond formation. Enzymes like XO and CYP also produce ROS during metabolic reactions, particularly in detoxification processes.^27^ Externally, ultraviolet radiation, pollutants, and ionizing radiation directly ionize cellular components, creating free radicals that propagate oxidative chain reactions. At low levels, ROS act as critical signaling molecules, modulating pathways such as mitogen-activated protein kinase (MAPK) and NF-κB to regulate cell growth, inflammation, and apoptosis. However, excess ROS oxidize lipids, proteins, and DNA, disrupting membrane integrity, inactivating enzymes, and causing mutations. Cells counteract ROS via enzymatic antioxidants: SOD converts O₂⁻ to H₂O₂, which CAT and GPX further break down into water. Non-enzymatic antioxidants like glutathione, vitamins C and E, and flavonoids directly neutralize radicals or repair oxidative damage. The Nrf2 pathway serves as a master regulator, translocating to the nucleus under oxidative stress to activate genes encoding detoxifying enzymes (eg, heme oxygenase-1) and antioxidants.^28^ Conversely, chronic ROS overproduction activates NF-κB, perpetuating inflammation and further ROS generation, a vicious cycle implicated in diseases. Mitochondrial ROS production is tightly linked to metabolic state; hypoxia or nutrient excess destabilizes the ETC, increasing electron leakage. Autophagy plays a regulatory role by degrading ROS-generating damaged mitochondria (mitophagy) or protein aggregates. Redox-sensitive cysteine residues in proteins act as molecular switches, altering function upon oxidation to regulate processes like cell differentiation and immune responses. However, irreversible oxidation leads to protein misfolding and aggregation, as seen in neurodegenerative diseases. ROS also influences epigenetic modifications, oxidizing DNA and histones to alter gene expression patterns. In cancer, ROS drives genomic instability and oncogene activation while paradoxically promoting survival pathways in adapted tumors. The duality of ROS, as essential messengers and toxic agents, hinges on precise spatiotemporal control, with imbalances leading to oxidative stress, aging, and pathologies. Thus, the mechanism of ROS intertwines generation, signaling, and scavenging, reflecting a dynamic equilibrium vital for cellular homeostasis.

### Redox adaptation in cancer cells

**Fig. 2:**
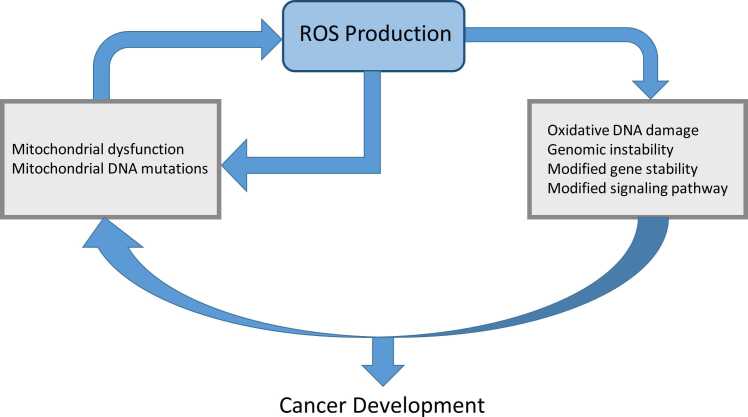
ROS has an essential role in cancer.

Cancer cells exhibit remarkable redox adaptability, reprogramming their antioxidant and pro-oxidant systems to survive oxidative stress while promoting proliferation, invasion, and therapy resistance. Possible factors contributing to the increased levels of ROS include stimulation of oncogenes, metabolic rearrangement, severe hypoxia in the tumor, and the modification of inflammatory and growth proteins.^29^ It was determined that when ROS levels are slightly elevated, they can interact with different proteins and intracellular signal transduction pathways that are linked to the survival and growth of tumors. The transcription factor Nrf2, often constitutively activated in cancers, drives the expression of antioxidant genes (HO-1, NQO1) and detoxifying enzymes, creating a buffer against ROS overload.^30^ In contrast, an abundance of ROS may eradicate tumor cells by triggering senescence and different ways of cell death, ie, autophagy, apoptosis, and ferroptosis. Cancerous cells upregulate the antioxidant system to maintain a homeostatic equilibrium of ROS within non-toxic thresholds. This mitigates the risk of senescence (cell aging) and apoptosis (cell death) and enables cells to take advantage of the proliferative benefits associated with high levels of ROS. Cancer cells use ROS to stimulate cellular proliferation and impede apoptosis by sustaining heightened intracellular ROS levels and triggering antioxidant defense systems. The role of ROS in the advancement of cancer is well recognized, with their level, duration, and distribution being crucial variables (Figure 2). There are two possible methods for treating cancer by focusing on the ROS system. One strategy involves using antioxidants to decrease the harmful impact of ROS, while the other involves intentionally increasing ROS levels to cause targeted death of cancer cells. However, both clinical and experimental studies have shown equivocal results regarding the validity of these concepts.^31^ Combining chemotherapy with molecularly targeted or immunological therapies yields superior outcomes in comparison to standard cytotoxic treatments, mainly due to its effective modulation of the immune system. Chemotherapeutic agents, such as platinum compounds and gemcitabine, cause oxidative damage to the DNA of cancer cells, resulting in the generation of substantial amounts of ROS as a component of their anti-tumor action.^32^ To improve the synthesis of proteins that induce apoptosis and decrease the synthesis of proteins that hinder cell death, a strategy involves boosting the production of death receptors and their binding molecules, as well as controlling the chemical modifications of Bcl-2 through ubiquitination and phosphorylation. Cancer cells also exploit NADPH, a critical reducing agent, by diverting glucose flux into the pentose phosphate pathway and upregulating NADPH-generating enzymes (IDH1, ME1). Autophagy and mitophagy are co-opted to recycle damaged organelles, preventing ROS leakage from dysfunctional mitochondria (Figure 2). Paradoxically, moderate ROS levels activate pro-survival pathways (PI3K/AKT, NF-κB, MAPK) that drive proliferation and angiogenesis.^33^ However, the capability of cells to adjust to oxidative stress, which is associated with many pathways that produce ROS, increases the resilience of cancer cells to chemotherapy. Studies have shown that tumor cells that are resistant to chemotherapy have elevated amounts of antioxidants, including CAT, GSH, and SOD.^34^ Additionally, CSCs maintain low ROS levels through enhanced FoxO signaling and ABC transporter activity, preserving their self-renewal capacity. Redox adaptation extends to metastasis, where ROS facilitate extracellular matrix degradation via MMP activation while antioxidants protect circulating tumor cells from oxidative assault in the bloodstream. However, this delicate balance creates vulnerabilities: pro-oxidant therapies (eg, elesclomol, arsenic trioxide) push adapted cells beyond their redox threshold, while inhibitors targeting Nrf2, glutathione synthesis, or thioredoxin reductase disrupt their antioxidant armor. Thus, cancer’s redox plasticity is a double-edged sword—enabling survival in hostile niches yet offering targets for precision therapies that exploit oxidative stress.

## Transcription factors and cellular redox system

**Fig. 3:**
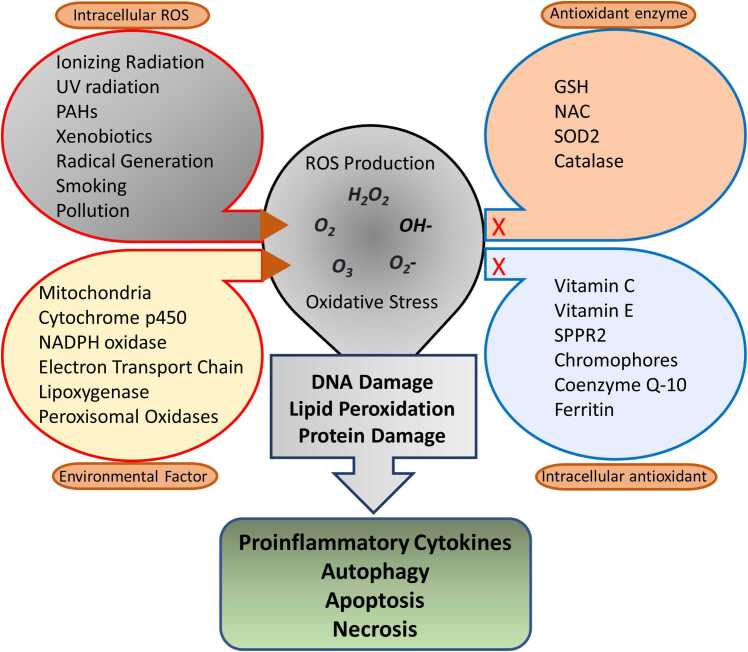
Oxidative stress and creation of ROS. Intracellular ROS and external stimuli (exogenous ROS) trigger the generation of ROS, causing oxidative stress. This, in turn, leads to the breakdown of DNA, lipids, and proteins, culminating in apoptosis, necrosis, autophagy, and the formation of pro-inflammatory substances.

ROS are essential for several cellular processes, including cellular proliferation, differentiation, migration, and apoptosis. Conversely, oxidative stress is linked to cancer, chronic inflammatory disorders, and neurological diseases. ROS have a significant role regulation of proteins involved in cancer formation or malignant growth in several signaling pathways connected with cancer (Figure 3). The production of disulfide bonds is primarily caused by the interaction between ROS and the residues of cysteine with the thiol group. These bonds can lead to certain alterations in proteins, which may either increase or inhibit their function. The NF-κB, HIF-1, MAPK, PI3K, NRF2, and p53 signaling pathways are controlled by ROS and play a crucial role in cancer.

### NF-κB

**Fig. 4:**
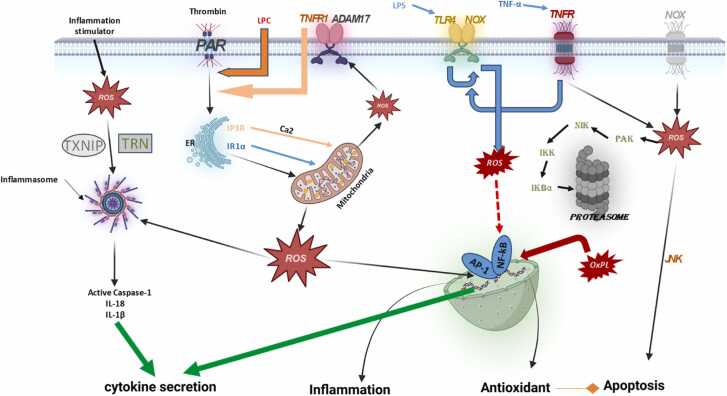
Regulation of oxidation-reduction (redox) processes in cells of the immune system.

**Fig. 5:**
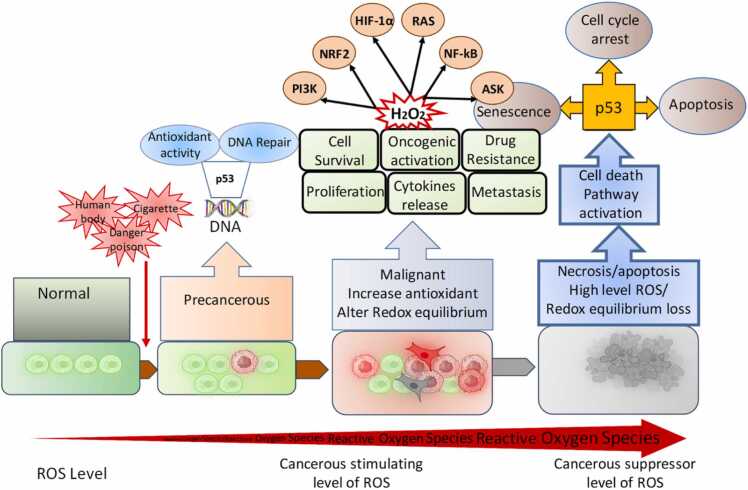
The relationship between mitochondria, ROS, and cancer may be described as a "vicious cycle." The malfunction or failure of mitochondria in cancer cells leads to the initiation of ROS, which in turn causes mutations in mitochondrial and nuclear DNA. These mutations limit oxidative phosphorylation (OXPHOS). Conversely, oncogenic ROS facilitates the progression of cancer by causing oxidative DNA damage and instability in the genome, altering gene expression, and contributing to many signaling pathways.

The NF-κB signaling system is implicated in several physiological processes, including inflammation and the immunological response. Furthermore, the NF-κB protein family has great medical importance since it is closely linked to inflammatory illnesses and the advancement and spread of cancer.^35^ The NF-κB pathway becomes active in cancer, resulting in resistance to apoptosis because it suppresses the antiapoptotic proteins. Additionally, this activation is correlated with heightened malignancy since it alters the transcription implicated in proliferation (cyclin D1 and Cellular Myelocytomatosis Oncogene), angiogenesis (vascular endothelial growth factor), and pro-inflammatory cytokines associated with cancer progression (Interleukin-1 and IL-6) (Figure 4). The basic action of transcription factors is linked to several forms of cancer, such as cervical, breast, colorectal, and acute myeloid leukemia.^36^ Activated NF-κB has been linked to several causes in these situations, such as bacterial or viral infections, inflammatory stimuli, DNA damage, radiation exposure, and increased amounts of ROS. ROS has shown a dual impact on NF-κB-mediated transcription, either facilitating or inhibiting it. The influence of ROS inside the cell is mostly dictated by the cell type or the location of ROS within the cell. The results suggest a complex and extensive connection between ROS and the NF-κB pathway (Figure 5). ROS-induced phosphorylation of p65 by PKAc, which is essential for its contact with CBP/300 and its role in gene expression regulation,^37^ exemplifies the stimulation of NF-κB by ROS. Furthermore, exogenous ROS, namely H_2_O_2_, may trigger the activation of NF-κB by causing phosphorylation of IkB, resulting in its breakdown in the proteasome. This process allows for the creation of dimerized NF-B proteins, which then go to the nucleus and commence the transcription of designated target genes. Furthermore, it was shown that H_2_O_2_ has an impact on phosphorylation and triggering of the IκB kinase (IKK) subunit, indicating that it hinders the function of an IKK phosphatase. The presence of H_2_O_2_ increases the process of combining two IKK/NF-κB Essential Modulator molecules, resulting in the deactivation of IKK and the initiation of the NF-κB pathway.^37^ Nevertheless, it has been shown that the suppression of NF-κB activity arises from many pathways. These include the deactivation of IkB by ROS-mediated glutathionylation. In addition, ROS can oxidize IKK kinase activity, resulting in its inactivation. ROS may also impede the proteasome, hence enhancing the stability of IkB. ROS-mediated activation of NF-κB involves the phosphorylation of IKK, leading to IκB degradation and NF-κB nuclear translocation. ROS-mediated inhibition of NF-κB involves the glutathionylation of IκB, preventing its phosphorylation and degradation, and thereby sequestering NF-κB in the cytoplasm. These distinct mechanisms highlight the dual role of ROS in regulating NF-κB activity, depending on the cellular context and the specific signaling pathways activated.^38^ Importantly, NF-κB can stimulate several genes, including XO, COX2, CYP, NOS-2, and NOX2 enzymes, to enhance the production of ROS during inflammation.^37^

### HIF-1

Hypoxia-inducible factor 1 (HIF-1) is a transcription factor that controls low amounts of oxygen. The heterodimer consists of alpha and beta subunits and is responsible for its operation. HIF-1 is produced continually, although hypoxia causes the buildup of HIF-1.^39^ Prolyl hydroxylases (PHD), using oxygen, append hydroxyl groups to two prolines on HIF-1 under normal oxygen conditions. The alteration targets HIF-1 for ubiquitination and subsequent elimination by the Von Hippel-Lindau complex. In hypoxic settings, HIF-1 complex stimulates the transcription that has hypoxia response elements in the nucleus.^40^ The HIF pathway has been widely researched in different cancers, due to its involvement in regulating the genes linked to angiogenesis, cellular migration, metabolism, and glucose transport.^41^ ROS controls the HIF-1 signaling pathway by promoting the accumulation of HIF-1, together with O_2_. It has been shown that mitochondrial O_2_• regulates HIF-1 during hypoxia.^42^, ^43^ Furthermore, it has been shown that HIF-1 remains stable under normal oxygen conditions when exogenous H_2_O_2_, glucose oxidase, or SOD2 knockdowns are introduced. This method includes both oxygen radicals (O_2_•) and H_2_O_2_. The suggested approach is the direct suppression of Prolyl hydroxylases activity, most likely accomplished by oxidizing its Fe2+ component.^40^ The production of H_2_O_2_ is generally recognized as a need for growth factor receptor signaling, primarily involved in the regulation of protein phosphorylation. This susceptibility is related to the existence of an acidic cysteine residue in the active region of protein tyrosine phosphatases. The phosphorylation of a cysteine in the active site by casein kinase-2 or the oxidation of that cysteine by H₂O₂ inactivates Phosphatase and Tensin Homolog through oxidation, leading to increased PI3K/Akt signaling and promoting cell survival and proliferation. Thioredoxin reduces the oxidized Phosphatase and Tensin Homolog, restoring its activity and thereby downregulating the PI3K/Akt pathway. This redox-dependent modulation is crucial for balancing cellular processes and preventing aberrant signaling that could lead to diseases such as cancer.^44^ This suggests that redox regulation plays a crucial role in this signaling cascade. ROS has a critical role in the intracellular pH and the metastasis of lung cancer.^45^

### MAPKs

MAPKs, a category of serine/threonine kinases, are responsible for regulating differentiation, proliferation, migration, senescence, and apoptosis. MAPKs phosphorylate various intracellular substrates on stimulation, including membrane transporters, transcription factors, and nuclear pore proteins. MAPKs may be stimulated by growth hormones in response to external stimuli. The regulation of the RAS/MAPK pathway occurs at several levels, its initiation is strongly associated with the generation of ROS. Studies have shown that antioxidant treatment may prevent the activation of MAPK after the stimulation of growth factors in several experimental situations.^46^ In addition, the conversion of RAS has been linked to the stimulation of NOX1 via a route involving RAS-RAC1-NOX1.^47^ The presence of ROS may stimulate EGF or MAPK signaling, even in the lack of EGF, when exposed to oxidative conditions or stimuli.^48^ The redox sensors TRX/Apoptosis Signal-Regulating Kinase 1 (ASK1) within the MAPK cascade are expected to have the most comprehensive characterization. TRX inhibits ASK1's kinase activity by direct binding. The oxidation of TRX by ROS causes it to separate from ASK1, resulting in the activation of ASK1. Consequently, a threonine residue in the kinase domain of ASK1 is phosphorylated, resulting in the activation of c-Jun N-terminal Kinase and p38 signaling pathways.^49^ Moreover, it was shown that ROS may trigger ERK signaling, likely via activating the epidermal growth factor receptor (EGFR) situated upstream. Research within this context has shown that EGFR signaling initiates the activation of PI3K and the production of H_2_O_2_ via NOX.^50^ In addition, it was shown that ROS may support EGFR-mediated signaling by triggering the protease-dependent shedding of ligands in the plasma membrane or by deactivating the Src homology 2 domain-containing phosphatase-2 phosphatase.^51^, ^52^ Moreover, the activation of MAPK has been associated with changes in the mitochondria, which might enhance the generation of ROS inside the mitochondria. it was shown that phosphorylating and activating DRP1 in RAS-modified tumors results in mitochondrial fission, an augmentation in the number of mitochondria, and a reduction in ATP synthesis by mitochondria.^53^

### p53"

The p53 gene is the most often altered gene that suppresses tumor growth in human cancer. The production of a transcription factor might trigger cell cycle arrest, senescence, or cell death as a reaction to DNA damage. This transcription factor has a vital function in preventing the buildup of mutations that might cause cancer.^54^ P53 is also linked to further cellular processes like metabolism, autophagy, cellular adaptability, and the activation of additional networks that restrict tumor growth.^55^ P53 has been shown to promote gluconeogenesis, boost antioxidant activity, reduce lipid synthesis, and increase fatty acid oxidation, which are metabolic goals. In non-stressed cells. However, under certain stressful situations, p53 builds up in the cytoplasm and triggers the activation of the ROS-producing enzymes quinone oxidoreductase (NQO1), proline oxidase, Bcl-2-associated X protein, and p53 upregulated modulator of apoptosis. These enzymes cause the separation of mitochondria and the production of ROS, indicating to oxidative stress and programmed cell death.^56^ TIGAR, the TP-53-tempted glycolysis and apoptosis regulator, is a pivotal focus of the p53 protein, involved in regulating metabolism and managing ROS. TIGAR inhibits the glycolysis rate and facilitates the uptake of glucose into the pentose phosphate pathway, a major supplier of NADPH required for the depletion of GSH. Furthermore, it was shown that mutations in the p53 gene inside cancer cells result in a decrease in mitochondrial respiration and an elevation in glycolysis.^57^ The data presented suggests significant variations in the control of ROS in cancer cells with either normal or mutant p53, and likely in the cellular responses to ROS generation.

### NRF2

NRF2 is a regulatory protein that controls the activation of many genes involved in safeguarding cells from harm. These genes participate in activities such as detoxification, autophagy, apoptosis, proteolysis, xenobiotic metabolism, cell proliferation, and lipid and carbohydrate catabolism. NRF2 enhances redox homeostasis by increasing the production, expression, and redox cycling of thiol-based antioxidant enzymes and GSH, which is its most comprehensively characterized function. NRF2 selectively regulates the expression of antioxidant genes.^58^ Keap1 tightly regulates the activation of NRF2. Under conditions of oxidative stress, the cysteine residues in Keap1 undergo oxidation, resulting in a modification of the structure of the Keap1-NRF2 complex. After entering the nucleus, NRF2, that is not attached to anything, starts the process of activating genes that protect cells, such as resistance-linked protein, autophagy, antioxidants, apoptosis-regulated genes, and glutamate-cysteine ligase (GCL), by attaching to the antioxidant response element. NRF2 was originally characterized as a tumor suppressor in recent studies^59^ to also promote the advancement of cancer, invasion, metastasis, and resistance to chemotherapy. The potential of NRF2 to remove chemoresistance produced by ROS makes it an intriguing target. Exposure to electrophilic compounds or ROS leads to alterations in cysteine residues on KEAP1. Leading to a rapid elevation in NRF2 levels. In contrast, under normal, non-stressed circumstances, the levels of NRF2 are minimal.^60^ Other mechanisms for the breakdown of NRF2 include the interaction between the nuclear protein transducin repeat-containing protein and phosphorylated NRF2 and CUL1, which leads to the ubiquitination of NRF2.^61^ Additional processes include the breakdown of Keap1 facilitated by phosphorylated p62/SQSTM1, as well as changes that occur after protein synthesis, such as sumoylation, acetylation, or ROS-induced hypomethylation of the NRF2 promoter.^48^ Cancer cells often exhibit abnormal activation of NRF2, which is not surprising given that malignancy is commonly linked to significant antioxidant, drug-detoxifying, and cytoprotective cellular activities.^62^

Increased expression of NRF2 has been associated with several types of cancer, including tumors that are resistant to chemotherapy and have poor prognoses.^63^ The heightened NRF2 activity is linked to a multitude of activities that function at different levels. Keap1/NRF2 pathway is associated with genetic alterations that contribute to the development of different types of cancer. The first cell lines harboring Keap1 mutations were obtained from human lung cancer. Changes in the Keap1 gene have been detected in a growing range of tumors,^64^ alterations activate NRF2 and protect cells against oxidative stress. SiRNA may be used to suppress the expression of NRF2 in gallbladder cells lacking Keap1, hence increasing the susceptibility to 5-FU.^65^ Similarly, the Keap1 genetic mutation led to a reduced clinical response to platinum-based medication treatment and was associated with the activation of the NRF2 pathway.^66^ The occurrence of somatic mutations in NRF2 is much less than in comparison to Keap1. In addition, it has been associated with esophageal squamous cell carcinoma,^67^ lung cancer,^68^ and HCC.^69^ The carcinogenic alleles Kirsten Rat Sarcoma Viral Oncogene Homolog (KRAS), B-Raf Proto-Oncogene, Serine/Threonine Kinase, and Cellular Myelocytomatosis Oncogene might potentially activate NRF2 transcription.^70^ The upregulation of oncogenic KRAS in NSCLC induces an elevation in NRF2 mRNA levels via the KRAS-ERK signaling pathway, resulting in resistance to cisplatin. The inhibition of NRF2 in these cells may be achieved by the use of RNA interference (RNAi) or by using the NRF2 inhibitor bristol.^71^ Moreover, the upregulation of PALB2 in breast and pancreatic cancers results in a reduction in ROS levels and promotes the accumulation of NRF2 in the nucleus.^72^ iASPP, a well-known antagonist of the p53 protein, has shown the ability to diminish the degradation of NRF2 via the ubiquitin-proteasome system. This is accomplished by binding to Keap1 via a DLT motif. Therefore, iASPP facilitates the formation and maintenance of resistance to chemotherapy in cancer cells.^73^

### Additional transcription factors

Several transcription factors are involved in chemoresistance mediated by ROS. NF-κB transcription factors play a crucial role in the advancement of cancer and the emergence of resistance to treatment. Manganese SOD is a key antioxidant that is regulated by NF-κB.^74^ It can protect against cell death prompted by oxidative stress. Recent findings indicate that anthracyclines, taxanes, and platinum-based medicines, along with other chemicals, may stimulate NF-κB pathway in pre-clinical animals. The stimulation of subsequent mediators, such as genes that prevent cell death, is essential in the development of resistance to chemotherapy.^75^ Inhibiting NF-κB activity may trigger apoptosis and reduce resistance in certain cancer cells.^76^ Studies have shown that sulfasalazine, which inhibits the NF-κB pathway, increases the sensitivity of cancer cell lines to gemcitabine, a chemotherapeutic medication used to treat pancreatic cancer.^77^ it was shown that MiR-146a-5p improves the effectiveness of gemcitabine in eliminating pancreatic ductal adenocarcinoma cells by modulating the HMGB1/TLR4/NF-κB/P-gp pathway.^78^ I-B, bortezomib, curcumin, and fisetin are examples of NF-κB inhibitors that shown significant promise in addressing chemoresistance in cancer treatment. Peroxisome Proliferator-Activated Receptor Gamma Coactivator 1 (PGC-1) is a recognized controller of mitochondrial biogenesis and oxidative metabolism, as well as a coactivator involved in transcription. The involvement of ROS in metabolic reprogramming has been linked to its function in promoting resistance to chemotherapy in tumors. Suppressing oxidative phosphorylation or downregulating PGC-1^79^ may enhance the vulnerability of cisplatin-resistant NSCLC cells.

Furthermore, Sirtuin 3 boosts the capability of colorectal cancer cells to survive chemotherapy by improving the PGC-1 expression and stimulating the antioxidant function of SOD2. Blocking Sirtuin 3 results in the decrease of PGC-1 expression, which enhances the susceptibility of cancer cells to chemotherapy.^80^ FOXO transcription factors also modulate the expression of antioxidant enzymes.^81^ Furthermore, ROS regulates the activity of FOXO, which is essential in the interaction relating ROS and the transcription factors for FOXO. FOXO3. protects against the harmful influences of doxorubicin and is linked to heightened PI3K/Akt activity in a cell line of chronic myelogenous leukemia.^82^ FOXO3 in glioblastoma cells mostly triggers the gathering of β-catenin in the cell nucleus, resulting in resistance to temozolomide.^83^ Although the direct role of managing ROS in the various phases of chemoresistance mediated by FOXO3 is uncommon, it presents a potential area for further investigation by examining the interaction involving ROS and FOXO3.^84^ The transcription factor HIF-1 has a role in the cellular response to hypoxia and is involved in the emergence of treatment resistance in several cancer types. ROS build-up in CRC stimulates HIF-1, which successively generates the release of 5-fluorouracil (5-FU) via the beginning PI3K/Akt signaling. The connection between HIF-1 and β-catenin mediates this process. Pharmacologically blocking HIF-1 or β-catenin might potentially reverse resistance to 5-FU in CRC by altering glucose metabolic reprogramming pathways.^85^ Moreover, the stabilization of HIF-1 is influenced by the interaction between NRF2 and HIF-1. The feedback loop between NRF2 and HIF-1 in hypoxic tumor environments creates a robust survival mechanism for cancer cells. This crosstalk enhances the antioxidant defense, promotes drug detoxification, and activates cell survival pathways, all of which contribute to chemoresistance. Understanding this interplay provides potential targets for developing therapeutic strategies to overcome chemoresistance and improve cancer treatment outcomes. The potential resistance to hypoxia in pancreatic and lung cancer cells might be achieved by disrupting the NRF2-HIF-1 axis.^86^

### ROS regulating stem cells

CSCs are a subset of cells inside a cancer that can resist therapy. Since their discovery four decades ago, they have undergone thorough examination due to their involvement in the resistance of tumors to treatment. The primary emphasis of the processes behind drug resistance in CSCs is in their ability to transition into dormant states initially and then resurface after an initial effective round of chemotherapy.^87^ The resistance of CSCs to chemotherapy is due to the existence of minimal amounts of ROS, which is a crucial property of CSCs controlled by the enhancement of mechanisms that scavenge free radicals.^88^ It was shown that CSCs have lower levels of ROS in comparison to cancer cells that have a high rate of proliferation. CD24-negative or low and CD44-positive breast CSCs exhibit greater resistance to radiation because of their reduced levels of radiation-induced ROS. It is important to mention that these minimal amounts of ROS are linked to CSCs such as CD13 and CD44. Studies have shown that stomach CSCs are protected against elevated ROS in TME via a modified form of CD44 called CD44v. CD44v can enhance the production of GSH by alleviating the cystine/glutamate substitute transporter xCT in gastrointestinal CSCs.^89^ CD44v amplifies the production of GSH, resulting in a rise in cellular antioxidant capability and facilitating significant resistance to chemotherapy. Gastrointestinal CSCs have increased amounts of ROS due to the depletion of CD44 by RNAi, making them more susceptible to existing cancer therapies.^90^ Head and Neck Squamous Cell Carcinoma cells that express CD44v have elevated xCT expression, heightened GSH levels, and reduced cellular ROS levels. RNAi is used to silence the xCT gene, facilitating the development of Head and Neck Squamous Cell Carcinoma CSCs in both laboratory conditions and in vivo individuals.^91^ The CD13 molecule in the CSCs has shown the capacity to have a suppressive influence on ROS, resulting in the development of resistance to chemotherapy. Liver cancer cells that are resistant to drugs exhibit elevated levels of CD13, a protein that governs the process of epithelial-mesenchymal transition induced by transforming growth factor-beta (TGF-β) and amplifies the generation of ROS stimulated by TGF-β.^92^ Moreover, the amplification of responsiveness to 5-FU and the breakdown of CSCs might potentially be accomplished by disrupting the ROS pathway via the suppression of CD13.^93^ Therefore, by diminishing the shielding function of the ROS scavenger pathway, CD13 and CD44v enhance the antioxidant capacity of CSCs by promoting the activity of the cystine/glutamate antiporter system xc⁻ and increasing glutathione synthesis. This enhanced antioxidant capacity enables CSCs to efficiently scavenge ROS and maintain redox homeostasis, contributing to their resistance to oxidative stress-inducing therapies. Targeting CD13, CD44v, or system xc⁻ could sensitize CSCs to oxidative stress and improve the efficacy of cancer treatments. Mounting data indicates that the signaling pathways linked to ROS have a substantial impact on the biology of CSCs. FOXO3a has a crucial role in controlling oxidative stress and is necessary for maintaining the features of CSCs. FOXO3a exhibited significant expression in leukemia-initiating cells in chronic myeloid leukemia. The survival of LICs depends on the presence of FOXO3a since it inhibits apoptosis. The findings suggest that the loss of FOXO3a substantially diminishes the capacity of LICs to induce myeloid leukemia in a mouse model that lacks FOXO3a.^94^ This finding provides evidence for the involvement of FOXO3a in the function of LICs. The research revealed that TGF has a pivotal function in controlling the activity of FOXO3a. Other signaling systems, in addition to TGF-, are also involved in the control of FOXO3. Studies showed that the PI3K-AKT pathway controls the preservation of stem cell-like properties and survival in a specific model of breast cancer via the FOXO-Bim axis.^95^

Studies have shown that NRF2 signaling is crucial for the preservation and resilience of CSCs. Enhancing the stability of NRF2 enhances the concentration of CSCs in breast cancer cells by regulating the shift from elevated levels of ROS to reduced levels of ROS.^96^ Studies have shown that NRF2 performs a similar role in glioma stem cells (GSCs). Moreover, the ability of GSCs to self-regenerate and develop tumors was greatly reduced when NRF2 was eliminated.^97^ Recent studies have shown that the activation of NRF2 signaling is essential for the maintenance of CSCs in several kinds of malignancies.^98^ The suppression of NRF2 activation limited the production of CSCs-like traits in ovarian cancer cells that had high levels of aldehyde dehydrogenase expression. Furthermore, a connection was shown between increased levels of p62 in liver cancer and breast cancer, as well as the ability of CSCs-like cells to withstand stress, which was facilitated by NRF2. Knocking down p62 led to a substantial decrease in both NRF2 levels and autophagy markers in CSCs.^99^ The existing information indicates that CSC adhesion molecules have a notable impact on the regulation of the defense against ROS. Targeting mechanisms related to ROS therapeutically show potential in eradicating therapy-resistant CSCs, since preserving depleted levels of ROS is crucial for resistant CSCs.^100^

### Modulating the TME by ROS manipulation

The ROS arbitrated the immunological and stromal cells in the TME is crucial for the development of chemoresistance and its direct impact on cancer cells.^101^ TME is characterized by hypoxia, which occurs due to a discrepancy between excessive oxygen use and insufficient oxygen delivery. HIF-1 overexpression plays a critical role in the emergence of therapeutic resistance due to hypoxia.^102^ HIF-1 stimulates glycolysis, leading to the formation of lactic acid, which causes the pH to become acidic. Acidifying the environment outside of cells could impede cellular immune responses, resulting in the development of resistance to drugs. Research has shown that hypoxia triggers cancer cells to produce an excessive amount of ROS, leading to changes that facilitate the development of resistance in tumor cells.^103^ Furthermore, stromal cells, such as immune cells, Cancer-Associated Fibroblast (CAF), and endothelial cells, have a substantial impact on the production of ROS, hence influencing their activation. Both factors may exacerbate drug resistance and lead to the onset of cancer.^104^

### ROS regulates the activity of fibroblasts that are involved with tumors

CAFs promote cancer growth and regulate drug response via multiple mechanisms. These mechanisms include the transmission of exosome vesicles, modification of tumor cell metabolism, and cytokine secretion that impact the tumor via paracrine mechanisms. Studies have shown that ROS has a role in the development of drug resistance, which is facilitated by CAFs.^105^ The Warburg effect proposes that cancer cells mostly depend on glycolysis, rather than oxidative phosphorylation, as their main source of energy. CAF cells use the "reverse Warburg effect" to generate energy via aerobic glycolysis while concurrently producing lactate. Tumor cells use lactate as a fuel source for oxidative phosphorylation to produce energy. Drug resistance arises due to the buildup of endogenous ROS via several adaptive metabolic processes.^106^ Prostate cancer cells exhibited a notable reduction in their responsiveness to chemotherapeutic medicines when cultured in the presence of CAFs or exposed to a CAF-conditioned medium specific to the prostate. The reduced susceptibility was ascribed to the heightened concentrations of GSH and the oxidative stress caused by CAFs, which counteracted the efficacy of the therapies.^107^ The release of exosome vehicles by CAFs can enhance chemoresistance. A study has shown that miR-522 secreted by CAF-derived exosomes targets and inhibits ALOX15 in cancer cells. This inhibition reduces the production of lipid hydroperoxides and lipid ROS levels, making cancer cells resistant to ferroptosis. The resulting ferroptosis resistance contributes to enhanced cancer cell survival, tumor progression, and therapeutic resistance. Targeting this pathway could provide new strategies for sensitizing cancer cells to ferroptosis-inducing therapies.^108^ CAFs are a diverse collection of cells that originate from several cell types and exhibit considerable variability.^109^ Myofibroblasts, which are a specific kind of CAFs seen in aggressive adenocarcinomas, are distinguished by their expression of smooth muscle alpha-actin. More precisely, elevated levels of ROS inside cancer cells have a crucial impact on the conversion of normal fibroblasts into myofibroblasts. There is a hypothesis that increased amounts of ROS generated by NOX4 in prostate cancer might potentially initiate the activation of prostate fibroblasts. The activation process is also facilitated by the signaling of TGF-β. Hence, the suppression of NOX4 in stromal cells leads to a reduction in the alteration of fibroblasts to myofibroblasts, which is facilitated by TGF-1.^108^ The NOX4-TGF-β axis drives the transdifferentiation of fibroblasts into myofibroblasts, which remodel the tumor stroma. This remodeling creates a supportive microenvironment for tumor progression, immune evasion, and therapeutic resistance. Understanding this axis provides potential targets for developing therapies that disrupt stromal remodeling and enhance the efficacy of cancer treatments.

It was shown that autocrine stromal cell-derived factor 1 stimulates the formation of CAF myofibroblasts via a process that relies on ROS.^110^ HER2-positive breast adenocarcinomas have shown the presence of accumulating stromal cell-derived factor 1, together with a hallmark of oxidative stress and a high concentration of myofibroblasts.^111^ There is a notion suggesting that prolonged oxidative stress has a substantial impact on the distribution of various subtypes of fibroblasts. Studies have shown that long-term antioxidant therapy can reverse this impact. This finding provides evidence for the significant involvement of ROS in the stimulation of CAFs. ROS not only stimulates the generation of myofibroblasts, but also influences the ability of CAFs to invade, their interactions with tumor epithelial cells, and their metabolic communication with cancer cells.^112^ ROS significantly impacts CAFs, which are known for their capacity to regulate ROS levels, hence promoting the development of an immunosuppressive TME. For instance, CAFs may stimulate the transformation of adjacent monocytes into myeloid-derived suppressor cells (MDSCs), which subsequently inhibit the growth of T lymphocytes. Additionally, the production of ROS is essential in inhibiting the activation of CAF-induced MDSCs. To increase the growth of T-cells, it is possible to diminish the inhibition of MDSCs induced by CAF by scavenging ROS via the reduction of indoleamine 2,3-dioxygenase or NOX2. Blocking NOX4 activity facilitates the entry of CD8+ T lymphocytes into tumors that have a high presence of CAFs, hence improving the effectiveness of immunotherapy.^113^

### ROS regulating immune cells

There is strong evidence suggesting that specific immune cells, such as MDSCs, dendritic cells (DCs), tumor-associated macrophages, tumor-associated neutrophils, natural killer cells, as well as T and B cells, have a significant impact on the development and progression of various types of cancer. ROS has a substantial influence on the makeup of immune cells and the immunological environment of tumors, hence altering the body's immune response to cancer.^114^ The development of immunological patience in TME generated by ROS is a major component that influences treatment resistance. A significant influence of ROS on the populations of immune cells. Optimal amounts of ROS is necessary for the accurate stimulation and variation of T-cells in a condition of normal physiological functioning. T-cells are a specific kind of immune cell that activates the immune system when they encounter antigens. They also have a beneficial impact on tumors inside the TME. ROS are essential for the proper functioning of T-cells and the activation of T-cell receptors. This activation results in the synthesis of H_2_O_2_ and O_2_, which subsequently impact subsequent signaling steps.^115^ ROS in Low amounts are necessary for the accurate stimulation and variation of T-cells under normal physiological settings since they stimulate the NFAT cells and facilitate the successive production of interleukin 2. ROS also has a role in causing both caspase-dependent and caspase-independent death of activated T-cells, which might result in the removal of peripheral T-cells.^116^

**Fig. 6:**
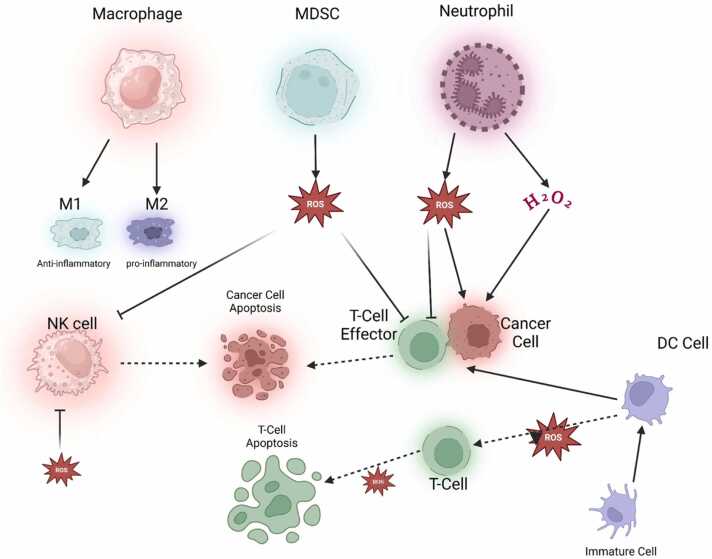
The inflammatory process is brought on by ROS and its schematic representation.

Furthermore, it was shown that increased levels of lactate generated by tumors trigger programmed cell death in inexperienced T lymphocytes via the creation of ROS, hence compromising the immune response to tumors. Targeting the network responsible for preserving a reservoir of immature T-cells may be advantageous in enhancing cancer treatment.^117^ Furthermore, an excessive abundance of ROS leads to a reduction in the levels of T-cell receptor and CD16 chain in T and NK cells. By inhibiting the activation of nuclear factor-kappa B, this hinders the synthesis of interferon, tumor necrosis factor, and interleukin-2. Moderate levels of mtROS enhance CD8⁺ T cell infiltration into the TME by promoting migration, adhesion, and metabolic reprogramming. High levels of mtROS lead to chronic oxidative stress, impaired mitochondrial function, and the upregulation of exhaustion markers, resulting in CD8⁺ T cell exhaustion. The dualistic role of mtROS highlights the importance of maintaining a balanced redox state in CD8⁺ T cells to optimize their anti-tumor functions. Targeting mtROS and metabolic pathways in CD8⁺ T cells could provide new strategies for enhancing the efficacy of cancer immunotherapies.^118^ Twenty years ago indicated that smoking has a substantial negative impact on the lifespan and effectiveness of NK cells. In contrast, several studies have shown the direct influence of smoking on NK cells, particularly in terms of its capacity to enhance the generation of interferon and other immune effector activity.^119^ A recent study indicates that ROS has a detrimental impact on the frequency and function of NK cells. For instance, the indoleamine 2,3-dioxygenase tryptophan catabolite L-kynurenine induces the programmed cell death of NK cells by activating the ROS pathway. A recent investigation into colorectal liver metastases has shown that increased levels of lactate in the TME indicate to the growth of mitochondrial ROS. As a result, this causes the liver-resident NK cells to die.^120^ Thioredoxin's stimulation of antioxidative pathways boosts the resilience of NK cells and adjacent T-cells against oxidative stress, possibly improving the effectiveness of immune treatment.^121^ ROS present in the TME also controls regulatory T-cells (Tregs), which are known for their capacity to inhibit anti-tumor immune activity by restricting the cytotoxic response of CD8+ T-cells. Macrophages have been shown to create ROS, which may induce the production of a significant number of regulatory T-cells (Tregs). Dexamethasone stimulates the production of ROS in macrophages, which in turn activates regulatory T-cells (Treg).^122^ Tumor-associated macrophages not only produce ROS in the TME, but they also create H_2_O_2_. This H_2_O_2_ suppresses the function of NK cells and cytotoxic T lymphocytes, subsequent in immunosuppression in the TME.^123^ Additionally, studies have shown that chemotherapy-induced ROS amplifies the immunosuppressive function of tumor-associated macrophages.^124^ The impact of ROS on macrophages cannot be categorized simply as either promoting or inhibiting cancer growth, owing to several circumstances, including the kind, stage, and therapy of the tumor. Neutrophil granulocytes are a kind of innate immune cell that demonstrates functional adaptability. Neutrophils, like macrophages, have a vital function in generating ROS inside the TME. Immunosuppression in the TME is induced by ROS produced by neutrophils, affecting several kinds of immune cells (Figure 6). Resting DCs primarily depend on oxidative phosphorylation powered by the tricarboxylic acid cycle to meet their energy needs. Activation of DCs and their pro-inflammatory activity relies on an increased degree of aerobic glycolysis, resulting in the formation of lactate when pathogen-associated molecular patterns (PAMPs) are stimulated. During glycolytic metabolism, significant amounts of inflammatory cytokines, nitric oxide (NO), and ROS are produced. Furthermore, studies have shown that the ongoing generation of ROS at minimal levels during the process of conventional DCs swallowing particles hinders the degradation of antigens by altering the pH of the phagosomes. The modification of pH is essential for efficiently delivering antigens to CD8 T-cells.^125^ The NRF2 is crucial in controlling redox activities in DCs and has been shown to suppress the proliferation of T-cells.^126^ In contrast, a reduction in NRF2 levels in DCs developed in heightened activation of helper T-cells and a decline in regulatory T-cells.^127^ Additionally, studies have shown that the polarization of the Th1/Th2 balance is affected by the GSH redox state in DCs.^128^ The abundant data demonstrate the vital significance of antioxidative stress systems in DCs. ROS have a substantial influence on the activation, death, and phenotypic differentiation of several immune cells. The appropriate initiation and maturation of NK and T-cells, as well as the normal functioning of antigen-presenting cells, depend on tightly controlled levels of ROS. Moreover, it enhances the capacity of neutrophils and macrophages to specifically locate and fight against tumors. However, regulatory T-cells and other myeloid cells, including macrophages and MDSCs, also use ROS to orchestrate immune suppression inside the TME.^129^ The immunosuppressive properties of ROS on T and NK cells might potentially function as a compelling resistance mechanism in the immune response. This leads to new questions about the improvement of anti-tumor systems. Furthermore, metabolic reprogramming might play a vital role in enhancing cancer immunotherapy.

## Therapeutic approach for specifically targeting ROS

Antioxidants and chemicals that hinder antioxidants have been suggested as potential treatments for cancer due to the dual function of ROS in both cancer development and resistance to chemotherapy. An extensive assessment conducted almost a decade ago indicated that around 45%-80% of patients diagnosed with breast cancer use antioxidant supplements in their treatment program. Antioxidants include vitamins C and E, multivitamins, antioxidant blends, glutamine, GSH, soy isoflavones, and melatonin.^130^ Antioxidative phytochemicals, whether ingested via diet or used as medications, have shown efficacy in cancer prevention rather than directly impeding tumor proliferation. This is consistent with the well-established cancer properties of ROS in the first phases of cancer formation. A study was done on a cohort of 77,446 individuals to assess the possible correlation between dietary antioxidant consumption and the risk of acquiring pancreatic cancer. The findings revealed an inverse relationship between dietary selenium consumption and the probability of pancreatic cancer occurrence.^131^ The therapeutic advantages or detrimental consequences of antioxidant supplementation in cancer therapy are now a subject of continuous discussion. Cancer treatment has included the use of ROS-induced cell death, ie, as Ferroptosis, necroptosis, and apoptosis, with chemotherapy and radiation. However, cancer cells acquire resistance to chemotherapy by activating their antioxidant pathways in response to oxidative stress. ROS modulators can eliminate enzymes that are responsible for antioxidant defenses. As a result, they have a considerable capacity to overcome resistance to therapy.^132^ The fundamental components of antioxidant systems are GSH, TrxRs, NRF2, SOD, and CAT, as previously mentioned. Moreover, several current clinical trials, mostly in phase I or II, are being conducted to examine the potential synergistic benefits of treatment based on ROS.

### Focusing on glutathione (GSH)

The natural compounds 3-bromopyruvate, sanguinarine, cucurbitacin B, and phenethyl isothiocyanate have shown anticancer effects in laboratory experiments, have a significant affinity for GSH.^133^ Increasing GSH efflux, conjugating with GSH, and oxidizing GSH with metals in the oxidation state or disulfides are all techniques to decrease intracellular GSH levels. APR-246 is the first pharmaceutical now being evaluated in clinical trials, which selectively binds to cysteine present in p53 and GSH. APR-246 had an additional anti-tumor impact on ovarian cancer cells when used in combination with cisplatin and doxorubicin. This effect was achieved by lowering the levels of free GSH inside the cells.^134^ APR-246 has shown effectiveness when used in combination with gemcitabine and docetaxel. Multiple ongoing clinical studies are assessing the safety and effectiveness of APR-246, either as a standalone treatment or in conjunction with anticancer medications, for various forms of drug-resistant malignancies. These trials are predicated on promising outcomes from preclinical investigations. APR-246 was provided either in conjunction with azacitidine or as a monotherapy in phase III. The effectiveness of an alternate nanoparticle, linked to disulfide-mediated GSH decrease, in successfully overcoming cisplatin resistance in ovarian cancer has been quite impressive. The GSH-sifting nanoplatform, consisting of poly (disulfide amide) polymers with a density of disulfide bonds, for the intravenous delivery of Pt prodrugs. Due to the absence of transit via the mononuclear phagocyte system, there was a negligible buildup of NP in the lung, spleen, and liver. The in vivo cytotoxicity of Pt was enhanced due to the rapid response to GSH and the fast rate of tumor development, resulting in the depletion of GSH.^135^ Recently, it was also shown that the new microwave-chemo-immunostimulant reduces ROS and depletes GSH in the TME. This altered redox microenvironment facilitated the conversion of pro-tumor M2 macrophages into anti-tumor M1 macrophages.^136^ Moreover, an increase in GSH outflow results in a decrease in intracellular GSH. The promotion of GSH efflux is facilitated by the modification of MRP1, which belongs to the family of adenosine triphosphate-binding cassette transporters. Verapamil, a calcium channel blocker, has been shown to have the ability to alter MRP1 and enhance the extrusion of GSH. This leads to the induction of death in multidrug-resistant cells.^137^ Studies have shown that some flavonoids, particularly apigenin and chrysin, have a strong ability to stimulate MRP1. This has significant possibilities for treating cancer cells that have an excessive amount of MRP1 and are unresponsive to chemotherapy.

### Inhibition of GSH production

Glutamate cyclase and GPxs are essential chemicals and enzymes required for the production and restoration of GSH. BSO is the only inhibitor of GCL that can inhibit GSH production used in clinical research. Emerging evidence suggests that BSO might play a crucial part in overcoming cisplatin resistance, improving the sensitivity of multidrug resistance in cytotoxic drugs.^138^ BSO significantly enhanced the efficacy of anticancer medications such as etoposide, doxorubicin, and vincristine in prostate cancer, which had developed resistance to these medicines.^139^ In addition, the levels of GSH are reduced and the levels of ROS are increased in high-risk neuroblastoma cell lines, ovarian cancer, and lung cancer when BSO is combined with a modulator of resistance-associated protein 1.^140^ BSO has been discovered to reverse antihormonal resistance in breast cancer metastasis by interfering with GSH's role in delaying apoptosis. A combination of BSO and melphalan at a dosage that is considered appropriate from a biochemical standpoint has been shown in many phase I studies, including cancer patients. Novel nanoformulation has been developed to effectively combat medication resistance in cancer cells while minimizing harmful effects on the body, known as systemic toxicity. Combining BSO with chlorin e6-loaded polymer NPs has been discovered to enhance the inhibition of cancer development by reducing GSH levels in mice with SCC-7 tumors. Another nanoparticle, which has been modified with folate, has been reported to effectively counteract carboplatin resistance in ovarian cancer cells. The key aspect of these nanoparticles that target folate is their capacity to selectively target diseased cells and decrease the accumulation of BSO in non-cancerous cells. This is significant since ovarian cancer cells have a higher expression of the folate receptor (FA-R) compared to normal cells.^141^ Valproic acid and indomethacin^142^ have shown suppressive activity in GCL and might potentially serve as novel chemotherapeutic sensitizing agents. The feasibility and effectiveness of VPA in combination with chemotherapeutics have been shown in clinical studies.^143^

### Targeting NRF2

The primary reason of chemoresistance is mostly due to the abnormal increase in the expression of NRF2. The responsiveness of cancer cells to certain chemotherapeutic drugs is significantly enhanced in vivo when NRF2 is suppressed by siRNA, shRNA, or RNAi.^144^ Several inhibitors of NRF2 have also shown the capacity to enhance chemosensitization. The powerful NRF2 inhibitor brusatol has shown the ability to halt the growth of pancreatic cancer cells and induce programmed cell death. Additionally, it enhances the anti-tumor properties of gemcitabine in pancreatic cancer cells by inhibiting the activation of NRF2 caused by gemcitabine.^145^ The combination of brusatol and cisplatin decreased the ability of cancer cells to form colonies and led to smaller tumor sizes in models of NSCLC that were transplanted into mice. However, when the function of KEAP1, a protein involved in regulating cell growth, was restored in lung cancer cells, additionally, anti-tumor impact of brusatol was nullified. Brusatol's effectiveness is associated with many types of cancer, including laryngeal squamous carcinoma and CRC.^146^ Surprisingly, it was shown that inhibiting the NRF2-TET1-AKR1C1 pathway, which is responsible for progestin metabolism, increases sensitivity of endometrial tumor cells to progestin therapy.^147^ Nevertheless, the bulk of the findings were limited to in vitro experiments. Hence, more investigation into the therapeutic use of brusatol is essential. A specific subdomain of NRF2 interacts with ML385, a probe molecule found by high-throughput screening, to suppress the expression of genes that are reliant on NRF2. ML385 had an increased effect on NSCLC cells in combination with platinum-covering medicines and other standard chemotherapy.^148^

Similarly, it has been suggested that the HTS-identified compound IM3829 improves the ability of radiation in lung tumor cell lines by inhibiting the binding of NRF2 and reducing target genes expression.^149^ Periplocin is considered a novel NRF2 inhibitor and is classified as a member of the cardenolide family of natural cardiac glycosides. Periplocin treatment effectively restored the resistance of gemcitabine-resistant pancreatic cancer cells to gemcitabine via regulating the signaling pathways and redox state controlled by NRF2.^150^ Moreover, tangeretin,^151^ camptothecin, wogonin, hederagenin, cryptotanshinone, luteolin, and chrysin have shown the ability to enhance the production of ROS in certain drug-resistant cancer cells by inhibiting NRF2. However, because of the absence of direct targeting of NRF2 medicines, issues arise about their shortage of toxicity and fussiness. Identifying agents that precisely target NRF2 is of utmost value.^58^ Potential advancements in therapeutic methods that focus on NRF2 may be achieved via the use of targeted protein degradation and molecular adhesives.^152^

### Targeting ROS modulators

In addition to the previously stated ROS regulators. For example, it was shown that ROS generated by NOX contributes to the development of drug resistance. Small molecule NOX inhibitors, such as GKT137831 (NOX4 inhibitor), NOS31 (NOX1 inhibitor), and capsaicin (NOX inhibitor), could enhance anticancer drug efficacy.^153^ Mitochondria, a substantial producer of ROS, might potentially be used to treat cancers that resist traditional therapy. Novel treatment approaches are now being investigated to address cancer chemoresistance by targeting mitochondrial transplantation, Ca2+ homeostasis, and mitochondrial metabolic pathways.^154^ The anti-diabetic drug metformin especially focuses on inhibiting mitochondrial complex I. Multiple studies have shown its potential to stun cancer cells resistance to 5-FU, doxorubicin, and cisplatin. Contrary to expectations, the administration of metformin did not enhance the outlook for individuals with cancer.^155^ Currently, several ongoing clinical trials are investigating the impact of metformin on different types of tumors, either as a standalone treatment or in combination. Individuals diagnosed with breast cancer participated in a clinical trial to assess the metformin effect compared to a placebo. The group that received metformin treatment did not demonstrate any noticeable improvement.^156^ Hence, it is essential to intensify efforts in repurposing antidiabetic medications for the goal of treating cancer. Alpha-tocopheryl succinate, fenretinide, and resveratrol are inhibitors of complex II, III, and IV, respectively, showed significant anticancer action. They are effective even in cancer cells resistant to chemotherapy.^157^ The Food and Drug Administration has approved the use of elesclomol, a medication specifically targeting mitochondria, for metastatic melanoma treatment.^158^ In a clinical investigation, patients with stage IV metastatic melanoma who were treated with a combination of elesclomol and paclitaxel every week saw a median progression-free survival that was twice as long as those who were treated with paclitaxel alone. This difference was statistically significant. However, people with advanced melanoma, regardless of their selection criteria, did not provide this significant discovery.^159^ Recent reports indicate that the efficacy of chemotherapy in treating tumors is enhanced by a novel nanoparticle called monocarboxylate transporter-targeting hybrid lipid-matter-coated silica carbon. From a mechanistic standpoint, lipid-matter-coated silica carbon nanoparticles significantly enhanced the production of ROS in mitochondria. This process results in the oxidation of nicotinamide adenine dinucleotide (reduced), leading to a limitation in ATP synthesis. This impaired the functionality of drug efflux pumps by shrinking and modifying the distribution of P-gp efflux pumps, thereby impairing the capacity of drug efflux pumps to expel pharmaceuticals. The inherent mitochondrial ROS burst was shown to be enhanced by another mesoporous silica nanoparticle. This sustained high oxidative stress enhances the susceptibility of the cancer cells to hypoxia-resistant photodynamic therapy.^42^ ROS is generated because of the strain exerted on the ER. ER stress is a state that arises from the unfolded protein response (UPR), whereby there is an accumulation of misfolded or unfolded proteins in the ER.

Another effective approach to treating cancer involves specifically addressing the oxidative damage resulting from ER stress. The three primary stress sensors in the UPR are inositol requiring protein 1 (IRE1), protein kinase R (PKR)-like endoplasmic reticulum kinase (PERK), and activating transcription factor 6 (ATF6). Ribonuclease inhibitors, such as MKC3946, STF-083010, and 8866, which specifically target IRE1, have been shown to improve chemotherapeutic agents' efficacy in many kinds of tumors.^160^ Furthermore, in cancer xenografts that are resistant to treatment, the ability to silence PERK in a controlled manner reestablishes the oxaliplatin pro-apoptotic effects. GSK2656157 and GSK2606414, which are new inhibitors of PERK, have been shown to significantly suppress cancer development and reduce vascular density in several human cancer.^161^ In addition, GSK2656157 enhanced the sensitivity of colon cancer cells to 5-FU treatment.^162^ One other method to improve the efficacy of anticancer agents is to interfere with ATF6. The sensitivity of U2-OS cells, which are a type of human bone osteosarcoma cells, to ER stress treatment is regained by Ceapin-A7, can targets ATF6 signaling.^163^ Furthermore, it was shown that hypoxia and processes reliant on the UPR collaborate to enhance resistance to chemotherapy. YC-1,^164^ dutasteride,^165^ and emetine^166^ are HIF inhibitors that can target HIF at various stages of a signaling system. These inhibitors have demonstrated therapeutic potential as standalone treatments or in combination with other cancer therapies.

A new chemosensitizing method has been introduced, which effectively targets both the UPR and HIF-1. ER stress and HIF-1 ubiquitination may be triggered by the HSP90 inhibitor geldanamycin. Within osteosarcoma cells, the chemoresistance is diminished by the presence of a different HSP90 inhibitor known as 17AAG.^167^ The signaling pathway of the mammalian target of rapamycin (mTOR) and ER stress has a mutual interaction, which might potentially have therapeutic implications.^168^ Rapamycin has been shown to induce irreversible ER stress, sustained autophagy, and reduced tumor development in a Kras/p53 mutant LC.^169^ Suppressing mTOR signaling has shown the ability to decrease cancer's susceptibility to chemotherapy while also inducing ER stress. These supplementary routes include the reversal of epithelial-mesenchymal transition, reduction in the expression of CSCs markers, and induction of mitophagy. It is noteworthy that metformin enhances the effectiveness of chemotherapy in fighting cancer by targeting mTOR pathway.^170^ Everolimus, a significant mTOR inhibitor, has shown efficacy in reducing tumor growth and overcoming resistance to chemotherapy in cancer patients. The US FDA has approved its use for the treatment of various cancers, such as metastatic hormone receptor-positive breast cancer and progressive, well-differentiated, non-functional neuroendocrine tumors in the gastrointestinal or pulmonary systems. These cancers must be unresectable, locally advanced, or metastatic. Several ongoing clinical trials investigating the efficacy of mTOR inhibitors, either in combination of Everolimus and paclitaxel, demonstrated promising clinical efficacy in patients with metastatic urothelial carcinoma who were not eligible for cisplatin, as shown in phase II open-label research.

### ROS generation by anticancer medications during cancer treatment

Several studies have shown that chemotherapy medications may induce oxidative stress in cancer patients.^4^ Nevertheless, there exists a very notable correlation between increased oxidative stress and the impacts of natural anticancer substances such as sesquiterpene-lactone-parthenolide. A distinguished phenolic compound obtained from hesperidin has been shown to combat colon cancer by inducing the production of ROS and triggering apoptosis via both intrinsic and extrinsic routes.^171^ ROS signaling is a crucial element in several phases of cancer cells, including survival, transcription, protein translation, tumor formation, and progression. The presence of ROS, such as hydrogen peroxide, leads to the programmed cell death known as apoptosis in cancer cells.^172^ Additionally, some anti-cancer medications can generate this compound, hence demonstrating an anti-cancer impact.^173^ study findings indicate that nimustine, actinomycin D, doxorubicin, mitomycin C, mitoxantrone, carmofur, gemcitabine, mercaptopurine, camptothecin, paclitaxel, vinblastine, and vinorelbine have the potential to induce substantial oxidative stress.^174^ Vinorelbine, a chemotherapeutic drug, reduces the levels of intracellular GSH and enhances the generation of intracellular ROS.^175^ Elevated concentrations of oxidants in the bloodstream have been seen in cancer patients after the treatment of epirubicin.^176^ Multiple anticancer medications induce DNA damage, leading to the eventual initiation of apoptosis. Epirubicin and doxorubicin can produce ROS,^177^ which may lead to DNA damage and ultimately exhibit anti-tumor effects.^178^ TAS-103 has anticancer activity via the induction of oxidative DNA damage.^179^ Vitamin C, also known as ascorbic acid, is a well-known antioxidant that plays a crucial role in preventing oxidative damage in cells. The mechanisms by which vitamin C prevents lipid peroxidation and reduces ROS levels include: Direct scavenging of ROS, regeneration of other antioxidants, chelation of metal ions, modulation of antioxidant enzymes, inhibition of pro-oxidant enzymes, protection of lipid membranes, induction of apoptosis in cancer cells. In addition to its antioxidant properties, vitamin C has been shown to induce apoptosis (programmed cell death) in cancer cells. This effect is thought to be mediated through the generation of hydrogen peroxide, which can selectively kill cancer cells while sparing normal cells. By inducing apoptosis, vitamin C can help eliminate malignant cells and reduce tumor growth. Eriocalyxin B, artemisinin, genipin, gemcitabine, spiclomazine, belinostat, artesunate, isoalantolactone, and dihydro artemisinin have been discovered to increase the production of ROS through different mechanisms, ultimately impeding the growth of cancer cells through ROS-mediated pathways.

## ROS as a double-game player

The antioxidant system found in a normal cell, consisting of enzymes such as GPxs, SOD, and CAT, together with the transcription factor Nrf2, helps maintain a balanced redox state even in the presence of ROS. The excessive creation of ROS disrupts the balance between antioxidants and pro-oxidants since the antioxidant defense mechanism is incapable of effectively removing the surplus. ROS effects in tumor cells have been recorded based on cancer development. Recent studies have emphasized the dual nature of ROS in malignant tissue. Increased production of ROS in a cancer cell initiates a response that helps maintain the equilibrium of redox reactions. Elevated expression of ROS contributes to the growth of cancer, the spread of tumors, the formation of metastases, and the survival of cells during the first phases of tumor growth.^180^ Elevated levels of ROS over the dangerous threshold lead to senescence, apoptosis, and cell death during the advancement of a tumor.^181^ The "dangerous threshold" of ROS levels was determined by a complex interplay of various factors. Dietary antioxidants, also known as phytochemicals, can regulate the level of cellular antioxidants. This, in turn, may influence the suppression of malignant cell development and induce cell death. The study demonstrated that the cytotoxicity caused by tamoxifen in breast cancer cells is regulated by the intracellular concentration of vitamin C. Vitamin C effectively prevented lipid peroxidation and decreased levels of ROS.^182^ In addition, resveratrol reduced the production of ROS in breast cancer cells, resulting in a reduction in cell death caused by paclitaxel.^183^ Vitamin E has been shown to decrease the production of ROS in an MCF-7 orthotropic breast cancer model, and this effect is dependent on the dosage. The study's results indicated a decrease in ROS levels during the treatment period, which was then followed by the growth of breast cancer cell tumors and an increase in p53 expression.^184^ The pro-oxidant properties of phytochemicals, including vitamin C, luteolin, apigenin, resveratrol, and epigallocatechin-3-gallate, are associated with elevated levels of ROS and cellular demise. An in vitro experiment has shown that higher doses of vitamin C may enhance pro-oxidant activity by increasing the generation of H_2_O_2_.^185^ Oxidative stress caused by Vitamin C may lead to a reduction in NAD levels and suppression of energy metabolism in breast cancer cells and colon cancer cells, ultimately resulting in cell death.^186^ Resveratrol demonstrated pro-oxidant action in the presence of copper ions, resulting in an elevation in hydroxyl radical generation.^187^ These phytochemicals are being extensively studied as prospective anticancer therapies to develop targeted death of tumor cells via the use of ROS.

## Conclusions and future perspectives

Cancer treatment is becoming more challenging due to adverse side effects and therapeutic resistance. ROS play a dual role in cancer, acting as both drivers of genomic instability, tumor progression, and metastasis, and as mediators of cancer cell vulnerability. Their dual nature stems from context-dependent thresholds: low ROS levels fuel pro-survival signaling, while excessive ROS triggers apoptosis or senescence. Consequently, researchers are focused on creating innovative cancer therapies. This review elucidated the significance of reactive species in cancer pathogenesis and the mechanisms by which cancer therapeutics target them. Numerous anticancer agents generate ROS and combat cancer via diverse mechanisms. Since most anticancer agents generate ROS in cancer cells, research should focus on mitigating their adverse effects. With the escalation of side effect concerns, a cancer cell-targeted prodrug may be developed to activate in response to elevated ROS in cancer cells, hence minimizing adverse effects. Nanoparticle-mediated redox-directed combinatorial anticancer treatment may be advanced by focusing on ROS formation. Alternative drugs have fewer therapeutic challenges than conventional methods, hence increasing their popularity. Certain bioactive food compounds generate ROS, induce oxidative stress, and eliminate cancer cells (ie, Curcumin, Quercetin, Capsaicin, etc).^188^, ^189^, ^190^ Improving the bioavailability of dietary components is the first step in using alternative medicines to increase ROS in cancer cells. Recent studies indicate that nanomedicine may facilitate the development of ROS-generating systems via both photodynamic and non-photodynamic approaches, therefore establishing photodynamic therapy as an anti-tumor agent. The analogous behaviors of tumor cell types indicate that different tumor types use the same pathways. It is more needed to focus on developing the most effective patient therapies. Certain studies have identified plasma-treated media, solutions, and water as beneficial and capable of eradicating cancer. In these cases, a carrier or method is required to maintain the efficacy of plasma-treated fluids. We must evaluate adverse effects and toxicity throughout treatment.

Advancements in ROS Modulation Techniques: Future research is likely to focus on developing more precise and effective methods for modulating ROS levels in cancer cells. ROS have been shown to play a significant role in the immune response. Future studies may explore how ROS modulation can be integrated with immunotherapy to enhance the body's immune response against cancer cells. This could involve strategies to induce immunogenic cell death that activates an immune response against dead-cell antigens and can enhance the efficacy of treatments such as chemotherapy and radiation by stimulating the immune system to target tumor cells and overcome the immunosuppressive TME which refers to the local environment surrounding a tumor that is modified by cancer cells to evade the immune system's attack, thereby promoting tumor growth and survival by preventing an effective anti-tumor immune response. Understanding these interactions could lead to new therapeutic strategies that target the TME to inhibit cancer progression and metastasis. As our understanding of the molecular mechanisms underlying ROS signaling in cancer improves, there will be a greater emphasis on personalized medicine. This involves tailoring ROS-based therapies to individual patients based on their specific cancer type, genetic makeup, and ROS profile. ROS modulation can be combined with other cancer treatments, such as chemotherapy, radiation therapy, and targeted therapy, to enhance efficacy and reduce resistance. There is a need for the development of new drugs and therapeutic agents that can effectively modulate ROS levels in cancer cells. This includes the discovery of small molecules, peptides, and biologics that can target specific ROS-generating or ROS-scavenging pathways. Future studies may focus on how ROS contributes to these processes and how it can be targeted to improve patient outcomes. By exploring these perspectives, future studies can provide a comprehensive overview of the latest advancements and potential therapeutic strategies involving ROS in cancer, ultimately contributing to the development of more effective and targeted cancer treatments.

## Funding and support

No funding.

## CRediT authorship contribution statement

**Dandan Tan:** Writing – original draft, Conceptualization. **Ning Ma:** Software, Resources, Investigation. **Yang Wang:** Writing – review & editing, Visualization, Validation, Investigation. **Xin Li:** Project administration, Investigation, Formal analysis. **Meiling Xu:** Writing – review & editing, Investigation, Conceptualization.

## Declaration of interest

The authors declare that they have no known competing financial interests or personal relationships that could have appeared to influence the work reported in this paper.

## Data Availability

Data will be made available on request.
